# Validation of a Fecal Glucocorticoid Assay to Assess Adrenocortical Activity in Meerkats Using Physiological and Biological Stimuli

**DOI:** 10.1371/journal.pone.0153161

**Published:** 2016-04-14

**Authors:** Ines Braga Goncalves, Michael Heistermann, Peter Santema, Ben Dantzer, Jelena Mausbach, Andre Ganswindt, Marta B. Manser

**Affiliations:** 1 Department of Evolutionary Biology and Environmental Studies, Animal Behaviour, University of Zurich, Winterthurerstrasse 190, 8057, Zürich, Switzerland; 2 Endocrinology Laboratory, German Primate Center, Kellnerweg 4, D-37077, Göttingen, Germany; 3 Department of Zoology, University of Cambridge, Cambridge, CB2 3EJ, United Kingdom; 4 Endocrine Research Laboratory, Department of Anatomy and Physiology, University of Pretoria, 0110, Onderstepoort, South Africa; Centre for Cellular and Molecular Biology, INDIA

## Abstract

In mammals, glucocorticoid (i.e. GC) levels have been associated with specific life-history stages and transitions, reproductive strategies, and a plethora of behaviors. Assessment of adrenocortical activity via measurement of glucocorticoid metabolites in feces (FGCM) has greatly facilitated data collection from wild animals, due to its non-invasive nature, and thus has become an established tool in behavioral ecology and conservation biology. The aim of our study was to validate a fecal glucocorticoid assay for assessing adrenocortical activity in meerkats (*Suricata suricatta*), by comparing the suitability of three GC enzyme immunoassays (corticosterone, 11β-hydroxyetiocholanolone and 11oxo-etiocholanolone) in detecting FGCM increases in adult males and females following a pharmacological challenge with adrenocorticotropic hormone (ACTH) and biological stimuli. In addition, we investigated the time course characterizing FGCM excretion, the effect of age, sex and time of day on FGCM levels and assessed the potential effects of soil contamination (sand) on FGCM patterns. Our results show that the group specific 11β-hydroxyetiocholanolone assay was most sensitive to FGCM alterations, detecting significant and most distinctive elevations in FGCM levels around 25 h after ACTH administration. We found no age and sex differences in basal FGCM or on peak response levels to ACTH, but a marked diurnal pattern, with FGCM levels being substantially higher in the morning than later during the day. Soil contamination did not significantly affect FGCM patterns. Our results emphasize the importance of conducting assay validations to characterize species-specific endocrine excretion patterns, a crucial step to all animal endocrinology studies using a non-invasive approach.

## Introduction

In their daily lives, animals often experience aversive stimuli (e.g. aggression with conspecifics, predator encounters, food restrictions, injuries, disease), which trigger a physiological stress response. In order to cope with such stressors, adrenocorticotropic hormone (ACTH) from the pituitary stimulates glucocorticoid (GC) production from the adrenal gland and its secretion into the bloodstream [1–4]. When produced in connection with short-term stress responses, GCs have beneficial and protective effects on the individual [5, 6] and thus, can positively influence its prospects of survival and reproduction (e.g.[7]). However, when production is induced for prolonged periods of time (i.e. chronically), GCs may negatively affect some physiological functions [8, 9] related to reproduction [10, 11], cognition [4, 12] and immune defense [5], which can result in reduced survival probability [13]. For these reasons, assessment of GC levels has become common practice for monitoring reproductive health and welfare of both wild and captive animals [14–17], particularly in threatened or endangered species (cheetah, *Acinonyx jubatus*:[18], wild dog, *Lycaon pictus*: [19], brown hyaena, *Hyaena brunnea*; [20], Western lowland gorilla, *Gorilla gorilla gorilla*: [21]). Consistently, a growing body of data has emphasized how GCs can be involved in the modulation of a wide variety of behaviors including anti-predator [22, 23], competitive [24], cooperative ([25] but see also [26, 27]), and affiliative behaviors [26], and thus can potentially have important effects on the display and maintenance of social relationships in animal societies.

GC levels can be measured directly from blood (plasma, serum) and saliva, or indirectly via assessment of their metabolite (GCM) concentrations excreted in urine and feces [28]. The choice of hormone matrix for sample analysis generally depends on the questions at hand, practicality of collection, the amount needed per sampling event, and the frequency of collection required [29]. While blood and saliva are most useful in studies investigating immediate changes in circulating GCs, urine and feces can be collected without direct contact and provide more integrative information on GC production, making those matrices better suited for the assessment of longer term variations in hypothalamic-pituitary-adrenal (HPA) axis activity [28].

The collection of feces for GC measurement presents several important benefits compared to other sample types: 1) feces are easy to collect, 2) can be collected once the subject has moved away from the site, allowing collection with minimum disturbance, 3) since feces collection does not influence the focal subject’s behavior, it allows for endocrine and behavioral data to be collected in parallel, and theoretically 4) an unlimited number of samples can be collected from each focal animal providing longitudinal information with flexible time frames. These benefits are particularly important when studying populations of wild animals, as they enable researchers to study the relationship between GC production and various ecological (e.g. rainfall, temperature, food availability) and socio-behavioral factors (e.g. social rank, aggression, communication, vigilance and response to predation etc.) within a natural setting [30].

Patterns, rates and paths of excretion as well as types of metabolites formed can differ substantially between species [31, 32], sexes [32–34], social rank [35–37], diet [38], season [39, 40], time of day [34, 41] and temperature [42]. It is thus essential to validate methodologies and understand the patterns of GC production and excretion for each studied species in order to generate biologically meaningful results [15, 39].

Meerkats (*Suricata suricatta*) are small, obligatory, cooperative breeding mammals that live in arid zones of southern Africa [43]. Groups are composed of a dominant pair and subordinate adults, sub-adults and juveniles of both sexes and varying degrees of relatedness [44, 45]. Females are philopatric [46], while males eventually disperse from their home territories in search of mates [47, 48]. Within a group, the dominant pair typically monopolizes reproduction, yet all group members contribute to the raising of the offspring, take turns looking out for predators, and contribute to burrow renovation [49–51]. Thus, meerkats present an ideal study system in which to assess links between GC and a variety of behaviors that are typical of animal societies in general and of cooperative breeding species in particular. In addition, the social and curious nature of the species makes meerkats very popular with the wider public and they are thus, commonly found in zoos the world over. Understanding meerkat physiological response to stressors will therefore also enable better monitoring of individual health and welfare of captive colonies.

Previous studies have already emphasized the importance of FGCM measurements for better understanding meerkat biology (e.g. [22, 52]). In these studies, an assay using an antibody against corticosterone was applied to monitor FGCM output. Although this approach has been validated [52], it is unlikely that corticosterone is the major circulating glucocorticoid in this species. Several studies have shown that in the majority of mammals cortisol is predominant over corticosterone in circulation (e.g. [53–55]), exceptions being some rodent species in which corticosterone predominates (e.g. [56, 57]). There is thus good reason to believe a priori that cortisol is also the main glucocorticoid produced by the adrenal gland in meerkats. In support of this, several studies have demonstrated that blood cortisol in the species is responsive to conditions in the environment indicative of ‘stress’ and that levels of cortisol are related to a number of ecological, social, or individual-state variables in meerkats [22, 25, 26, 58, 59]. It is therefore conceivable that in meerkats FGCM measurements based on the analysis of metabolites originating from cortisol may provide a more sensitive measure of adrenocortical function than the measure of corticosterone immunoreactivity previously used [52].

Therefore, the overall aim of our study was to evaluate a new test system for monitoring FGCM alterations in male and female adult meerkats. More specifically, we aimed to 1) determine stress-related physiological responses in meerkat feces by performing an adrenocorticotropic hormone (ACTH) stimulation test, 2) evaluate the suitability of the previously used corticosterone assay [52] by comparing its performance with that of two group-specific assays that measure metabolites of cortisol, 3) characterize the metabolites measured by the different assays using HPLC analysis, 4) determine the time course of FGCM excretion, 5) test the validity of the most reliable assay to detect FGCM increases following social stimuli, and 6) evaluate the potential impact of soil contamination (sand) of the feces upon FGCM excretion patterns. Using a captive meerkat colony, we conducted these validations in a controlled environment that allowed us to collect multiple daily samples per individual and to assess potential effects of age, sex, and time of day on FGCM excretion.

## Materials and Methods

### Ethics Statement

This experiment was done according to Swiss law, with ethical approval given by the relevant authority, *i*.*e*. the Swiss Animal Welfare Agency (permit numbers 30/2012 and 233/2014). No other permit was needed for handling the meerkats. This study did not involve any endangered or protected species. All details on sampling methods are given below.

### Animals and housing conditions

We collected fecal samples from male and female adult meerkats (age range: 11–58 months) housed at the University of Zurich in an outdoor-indoor enclosure (250m^2^). During the study, the colony was exposed to natural fluctuations in photoperiod and natural outdoor temperatures, although heat lamps are available both indoors and outdoors for extra warmth. Water was provided *ad libitum* and individuals were fed three times daily, with a mix of fruit, vegetables, mealworms, crickets, hard-boiled eggs and dead chicks. The captive colony originally started with four adults (1 female and 3 males) in 2011 and increased in size to 17 individuals throughout the time of the study. Information regarding the sexes, dates of birth and manipulation participation of all study animals is presented in Table 1.

**Table 1:** Individual ID, rank, sex and date of birth of all subjects used in the ACTH challenge tests I and II as treated individuals or as water-injected controls (water). F = female, M = male, ACTH I: April 2012, ACTH 2: May 2013.

ID	Rank	Birth	Challenge
ZIF001	Dominant	Septermber.2008	I & II
ZIM002	Dominant	July.2007	I
ZIM003	Subordinate	April.2008	I and water (II)
ZIM004	Subordinate	June.2008	I & II
ZIM005	Subordinate	May.2011	I & water (II)
ZIM006	Subordinate	July.2011	II
ZIF007	Subordinate	July.2011	II
ZIM008	Subordinate	July.2011	II
ZIF009	Subordinate	July.2011	II
ZIM010	Subordinate	February.2012	II
ZIM011	Subordinate	February.2012	II
ZIF012	Subordinate	February.2012	II
ZIF013	Subordinate	May.2012	II
ZIM014	Subordinate	May.2012	water (II)
ZIM015	Subordinate	May.2012	water (II)

### ACTH challenge

We conducted ACTH challenge tests in April 2012 (4 males, 1 female) and May 2013 (5 males, 5 females, Table 1). To establish basal FGCM levels we collected 1–3 fecal samples per day from each individual 5–9 days prior to the pharmacological challenge (pre-treatment levels). On the day of injection, we distracted the subjects by spreading mealworms on the ground and injected intramuscularly a single dose (5 IU per kg body weight) of synthetic ACTH (Synacthen Depot ampule 1mg/ml, Novartis Pharma Schweiz AG, Bern) on the thigh [52]. The focal individuals were well habituated to close human presence, which allowed ACTH administration without capture or restrain. On the day of injection and the three days thereafter, we attempted to collect three samples per day and individual. Subsequently, we collected two samples per day and study subject for up to two weeks after ACTH administration. Concurrently with the second ACTH challenge in 2013, we injected four males with distilled water (0.5ml/kg body weight) to assess the effect of the injection procedure on glucocorticoid production.

### Natural stressor: subordinate male eviction

To assess FGCM alterations in response to biologically relevant stressors, we opportunistically collected samples from 10 adult individuals (six males and four females) around the time of intense attack on one group member, which culminated in its permanent eviction from the group and relocation to a local zoo. In total, we collected 12 samples (from five individuals) prior to the observed aggressive interactions and 12 and 32 samples (from 10 individuals) within 48 h and 5 weeks after the observed attack, respectively. In addition, we collected fecal samples from the attacked male before and during the time when he was attacked.

### Fecal sample collection

In total, we analyzed 406 samples, which were collected while the animals remained in their group. Before each meal we fed each individual a food item covered with a unique combination of different colored indigestible glitter or chrome dioxide [60] and food dye to enable fecal sample identification and reliable assignment of samples to individuals. We inspected the enclosure for new feces every couple of hours and, if we did not find any samples from a particular individual, before the next meal, we gave it another food item with its respective glitter/food dye combination to improve our chances of collecting the aimed number of samples per individual per day. Upon collection, samples were placed into labeled plastic bags and stored at -20°C within 15 min. The material remained at -20°C until shipment (on dry ice) to the Endocrinology Laboratory at the German Primate Center for FGCM analysis.

### Hormone extraction and accounting for sample soil contamination

We processed and extracted all fecal samples following Heistermann et al. [61]. Briefly, samples were lyophilized for 72 h, pulverized, and 0.10–0.12 g of the fecal powder were extracted in 3 ml of 80% aqueous methanol by vortexing the suspension for 15 min. The applied protocol is strongly recommended by Palme and colleagues [53] who have shown that 80% aqueous methanol proved best suited for extraction of naturally occurring glucocorticoid (and other steroid hormone) metabolites from feces of virtually all mammalian species tested so far, yielding high recoveries (e.g. 80–90%) and precision [62–65]. Following extraction, we centrifuged the suspension, recovered the supernatant and stored it at -20°C until FGCM analysis [66].

Since feces were often deposited onto sandy ground, most samples were covered in a layer of soil. To minimize this “contamination” and thus aid in reducing non-biological variance in FGCM levels [67], we manually rubbed off the soil as best as possible before the samples were pulverized. We assessed the potential impact of the remaining sample “contamination” with soil on overall FGCM excretion patterns by determining in the samples from 2013 (n = 176 samples from 10 ACTH challenges, see “ACTH challenge” in Methods section) the amount of soil and subsequently comparing between fecal hormone concentrations (for both the CCST and 11β-hydroxyetiocholanolone EIA assays) uncorrected and corrected for soil content, i.e. expressed per fecal mass without soil (see [67]). Following centrifugation of the fecal suspensions for extraction (see above) and drying the fecal matter under a fume cabinet for >14 days, we separated the organic fecal material from the soil and determined a soil-free fecal dry weight for each sample. We then used this weight to calculate hormone concentrations per pure organic fecal mass.

### Steroid analyses

Fecal extracts resulting from the ACTH challenge in 2012 were measured for immunoreactive FGCMs using three different enzyme immunoassays (EIAs) detecting corticosterone (CCST) [68] and cortisol metabolites with a 5β-3α-ol-11-one structure (11oxo-etiocholanolone, [63] and 5β-3α,11β-diol-structure (11β-hydroxyetiocholanolone, [69]. The CCST EIA used the same antibody that had been used in the CCST RIA system validated for stress hormone assessment in meerkats by Young et al. [52]. Detailed information on antibody characteristics, standards, and hormone labels as well as on other assay details, e.g., data on assay sensitivities, are given in Heistermann et al. [68].

Fecal extracts resulting from the ACTH challenge II and water injections from 2013 were analyzed with the CCST and 11β-hydroxyetiocholanolone EIA only. This decision was based on the results from 2012 (see Table 2), which showed that 1) the values generated by the 11β-hydroxyetiocholanolone and 11oxo-etiocholanolone assays correlated strongly with each other for each of the 5 tested individuals (all r = 0.71–0.98; p < 0.0001) thus providing very similar response patterns to adrenocortical stimulation, 2) time lags of responses were identical for the two assays, but 3) peak increase in levels in response to the challenge was, on average, twice as large (13.6 vs. 6.1 fold) for 11β-hydroxyetiocholanolone than for 11oxo-etiocholanolone, indicating a higher sensitivity of the former to stressful events. The remaining fecal extracts from the subordinate male eviction event (see “Natural stressor: subordinate male eviction” in Methods section) were assayed only in the 11β-hydroxyetiocholanolone EIA, because the data from the two ACTH challenge tests as well as the control water injection tests indicated that this assay is more sensitive than the CCST assay for monitoring FGCM patterns in meerkats (see “ACTH challenge” in Results section).

**Table 2:** Fecal glucocorticoid concentrations in response to ACTH administration in individual meerkats measured using a 11β-hydroxyetiocholanolone, a corticosterone (CCST), and a 11β-oxo-etiocholanolone assay.

Animal	3α,11β-dihydroxy-CM				CCST				3α,11oxo-CM			
	Pre^b^	Peak^c^	Delta^d^	Lag^e^	Pre	Peak	Delta	Lag	Pre	Peak	Delta	Lag
ACTH 2012												
F1^a^	0.360	4.89	13.6	27	0.080	4.50	56.3	7	0.950	5.80	6.1	27
M2^a^	0.501	4.21	8.4	22	0.080	0.29	3.6	7	0.995	2.75	2.8	22
M3	0.294	0.90	3.1	70	0.055	0.05	0.9	—	0.585	2.04	3.5	70
M4	0.194	2.74	14.4	22	0.026	0.13	5.0	7	0.462	4.24	9.2	22
M5	0.244	17.0	70.8	22	0.068	2.88	42.4	7	0.348	16.4	46.9	22
**Median**	**0.294**	**4.21**	**13.6**	**22**	**0.068**	**0.29**	**5.0**	**7**	**0.585**	**4.24**	**6.1**	**22**
ACTH 2013												
F1	0.589	4.50	7.7	22	0.123	1.01	8.2	6				
F7	0.071	1.48	20.9	25	0.036	0.34	9.4	3				
F9	0.065	7.49	107.0	25	0.042	3.04	72.4	8				
F12	0.052	0.48	9.1	33	0.100	0.31	3.1	33				
F13	0.190	13.26	69.7	22	0.067	6.37	95.5	22				
M4	0.099	2.24	22.6	25	0.024	0.20	8.4	6				
M6	0.137	5.14	36.7	25	0.073	1.09	14.9	25				
M8	0.059	0.17	2.9	22	0.100	0.55	5.5	22				
M10	0.183	7.17	39.2	22	0.141	3.31	23.5	22				
M11	0.162	1.00	6.3	27	0.030	0.09	3.0	27				
**Median**	**0.118**	**3.37**	**21.8**	**25**	**0.064**	**0.78**	**8.9**	**22**				
Water injections												
M3	0.124	0.38	3.1	22	0.034	0.07	2.2	22				
M5	0.061	2.08	34.4	29	0.172	0.47	2.8	29				
M14	0.099	0.69	7.0	25	0.041	0.22	5.4	25				
M15	0.111	12.22	110.5	22	0.161	4.35	27.0	22				
**Median**	**0.105**	**1.39**	**20.7**	**24**	**0.101**	**0.35**	**4.1**	**24**				

^a^F = female, M = male,

^b^pre-treatment levels (mean) in μg/g feces (see Methods),

^c^peak levels in response to ACTH administration in μg/g,

^d^x-fold increase of peak levels above mean pre-treatment concentrations,

^e^lag-time in hours between administration of ACTH and peak CG response.

We performed all EIAs on microtiter plates according to the procedure described in detail by Heistermann et al. [68, 70]. Prior to steroid measurement, we diluted fecal extracts 1:10–1:1000 (depending on concentration and assay) in assay buffer. All samples were run in duplicate and samples with a coefficient of variation >7% between duplicates were re-measured. Serial dilutions of fecal extracts gave displacement curves parallel to those obtained with the respective standard in each assay. Intra- and inter-assay coefficients of variation of high- and low-value quality controls for each assay were <10% and <15%, respectively. All final hormone concentrations are given as μg/g fecal dry weight.

### HPLC analysis

To characterize the immunoreactive metabolites measured by the CCST and 11β-hydroxy-etiocholanolone assays, we performed reverse-phase high-pressure liquid chromatography (RP-HPLC) using the procedure previously described in detail by Möhle et al. [71] and Heistermann et al. [68]. We performed HPLC on a male and a female sample, each representing peak FGCM response to the ACTH challenge, to evaluate possible sex differences in the characteristics of excreted cortisol metabolites (c.f. [39, 72]). HPLC also enabled us to assess whether certain fecal androgen metabolites, which could potentially be detected by antibodies raised against cortisol metabolites [31, 68, 69], were co-measured by the two EIAs. We measured each HPLC fraction in the two aforementioned assays to generate the profiles of immunoreactivity.

### Data analysis

We generated composite (average) profiles of FGCM response to ACTH for the CCST and 11β-hydroxyetiocholanolone assay by calculating the percentage change in FGCM levels (relative to the mean pre-treatment baseline levels; set as 100%) across 8–12 h intervals following ACTH injection for each individual and averaging values across all individuals. We excluded M3, who clearly did not respond to the ACTH stimulation (see Table 2), from this and all other analyses.

We determined the time course of FGCM excretion as the lag time from the administration of ACTH (or water) to peak FGCM response, separately for each individual and assay. To investigate for a possible sex effect in FGCM response to the ACTH challenge, we compared the magnitude of peak FGCM elevation between sexes using a Mann-Whitney U-test.

To examine the effects of sex, age and time of day (morning (AM): 8-11am, midday (MD): 11:30-2pm, afternoon (PM): 3-6pm) on basal FGCM levels, we used all fecal samples collected during pre-ACTH challenge control periods (i.e. samples reflecting baseline FGCM levels), numbering 128 samples from 13 individuals. In this analysis, values were log-transformed to conform to assumptions of linearity, and linear mixed effects models (NLME package version 3.1–118 in R) were used to account for ACTH challenge (I and II) and individual identity as random factors, because two individuals were tested in both challenges (F1 and M4) and because we had multiple samples per individual which were not evenly distributed among the three day time periods.

The effect of the subordinate eviction on FGCM levels was assessed in multiple steps to maximize the information from our available data, due to uneven sample sizes between time periods. We used a Friedman test to assess overall individual changes in FGCM levels. Pre and post-attack “control” levels, and during- and post-attack attack levels were compared using Wilcoxon signed-rank-tests.

We determined the percentage of soil content of a sample by dividing the mass of soil per sample by the total mass of extracted fecal material. We assessed the correspondence between FGCM levels expressed per mass extracted feces and those expressed per “soil-free” fecal mass (see “Hormone extraction and accounting for sample soil contamination” in Methods section) by calculating Spearman rank correlations for each individual and separately for the CCST and 11β-hydroxyetiocholanolone assays. To examine whether the two assays differed in their degree of correspondence, we compared their correlation coefficients using the Wilcoxon signed-rank test. To further evaluate the influence of soil contamination on characteristics of FGCM pattern, we compared the magnitude of responses to ACTH (peak to baseline ratio) between uncorrected and “soil-corrected” FGCM profiles as well as the variability in pre-treatment FGCM baseline levels (calculated as coefficient of variation, CV) under the two conditions. Both comparisons were carried out using Wilcoxon signed-rank tests. All statistical tests, carried out in R Studio Version 0.98.1102 and SPSS (IBM SPSS Statistics for Macintosh, version 22.0), were two-tailed and the statistical significance level was set at 0.05.

## Results

### ACTH challenge

**Fig 1:**
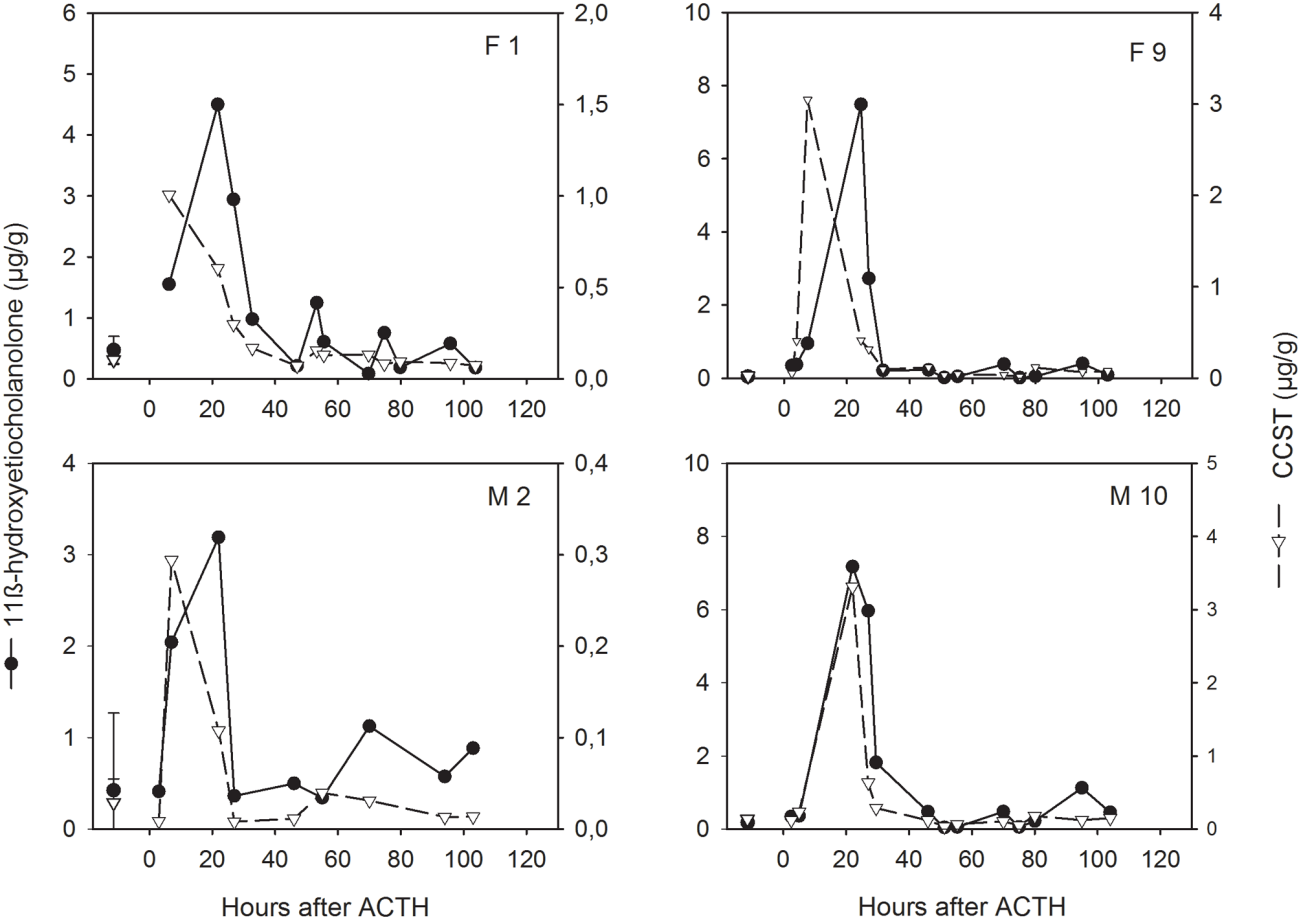
Representative profiles of immunoreactive 11β-hydroxyetiocholanolone (black circles) and CCST (white triangles) in two female (top) and male (bottom) meerkats after administration of ACTH at time 0. Data points before time 0 represent median ± SE concentrations (μg/g) of pre-treatment samples collected 5–8 days before ACTH injection.

In absolute terms, basal FGCM levels measured by the 11β-hydroxyetiocholanolone EIA were substantially (2–4 fold) higher compared to levels measured by the CCST assay (Table 2). In 14 of the 15 ACTH challenges conducted, we detected with both assays a strong response to the treatment denoted by a minimum 3-fold increase in FGCM levels (Table 2; Fig 1). The two assays, however, differed in their magnitude of the resultant increase, with 11β-hydroxyetiocholanolone presenting an overall median 17.7-fold peak response increase and CCST an 8.9-fold average increase (Table 2). In 10 out of the 14 cases, the increase measured by the 11β-hydroxyetiocholanolone assay was higher than that measured by the CCST assay (Table 2). For each assay, the magnitude of response varied between subjects (Table 2; see also Fig 1), but peak elevation in FGCM levels to ACTH administration was not statistically different between males and females for either assay (median ± SE fold increase in 11β-hydroxyetiocholanolone: males: 18.5 ± 8.1, females: 17.3 ± 16.7, U = 21, p = 0.755; CCST fold increase: males: 7.0 ± 4.8, females: 32.9 ± 16.0, U = 14, p = 0.228).

All four control individuals injected with water also showed an increase in immunoreactive CCST and 11β-hydroxyetiocholanolone levels in response to the treatment. For both measures, the increases were in the same range as those reported for the ACTH treated animals (Table 2). Similar to the findings of the ACTH challenges, the response was stronger for 11β-hydroxyetiocholanolone compared to CCST in all four cases (Table 2).

### Time course of FGCM excretion

**Fig 2:**
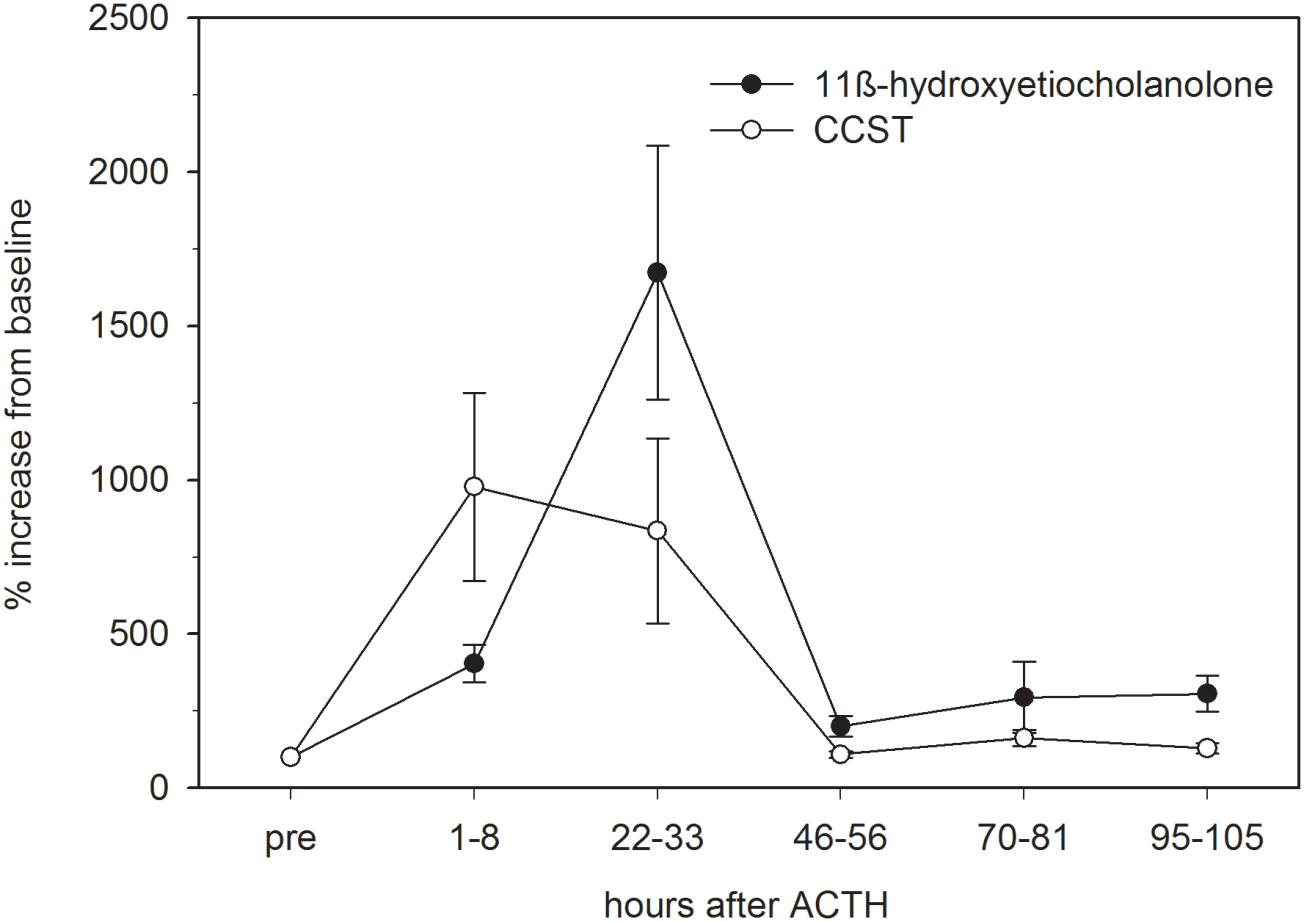
Percentage of response in immunoreactive fecal 11β-hydroxyetiocholanolone and CCST levels to ACTH administration in meerkats. Data points represent mean ± SE values calculated for 8-12-h intervals across the 14 individuals that responded to the ACTH challenge. Percentages were calculated in relation to pre-treatment baseline values (pre = 100%).

The timing of FGCM peak elevation varied between subjects and FGCM measure. Peak response was highly consistent across individuals for the 11β-hydroxyetiocholanolone assay, occurring on average 23.5 ± 0.9 hours (median ± SE, range 22–33 hours) after ACTH administration (Table 2, Fig 2), although levels already increased above baseline levels within 8 hours of the stimulation (see Figs 1 and 2). A lag time to peak response of about 24 hours (median ± SE: 24.5 ± 1.7, range: 22–29 hours) was also recorded in the four animals receiving water injections only (see “ACTH challenge” in Results section; Table 2). By contrast, the timing of the CCST response to ACTH was much more variable, with the peak elevation occurring between 3–8 hours in 8 cases and between 22–33 hours in the remaining 6 cases of the ACTH challenges (Table 2; see also Fig 1). In the four control animals that received water injections, the lag time ranged between 22–29 hours (Table 2). The composite profile depicted in Fig 2 demonstrates this overall temporal pattern in FGCM excretion and the differences between the two GC measures in this respect and shows that for both measures, FGCM levels had returned to pre-injection baseline values at about 40–50 hours (see also Fig 1).

### HPLC analysis

**Fig 3:**
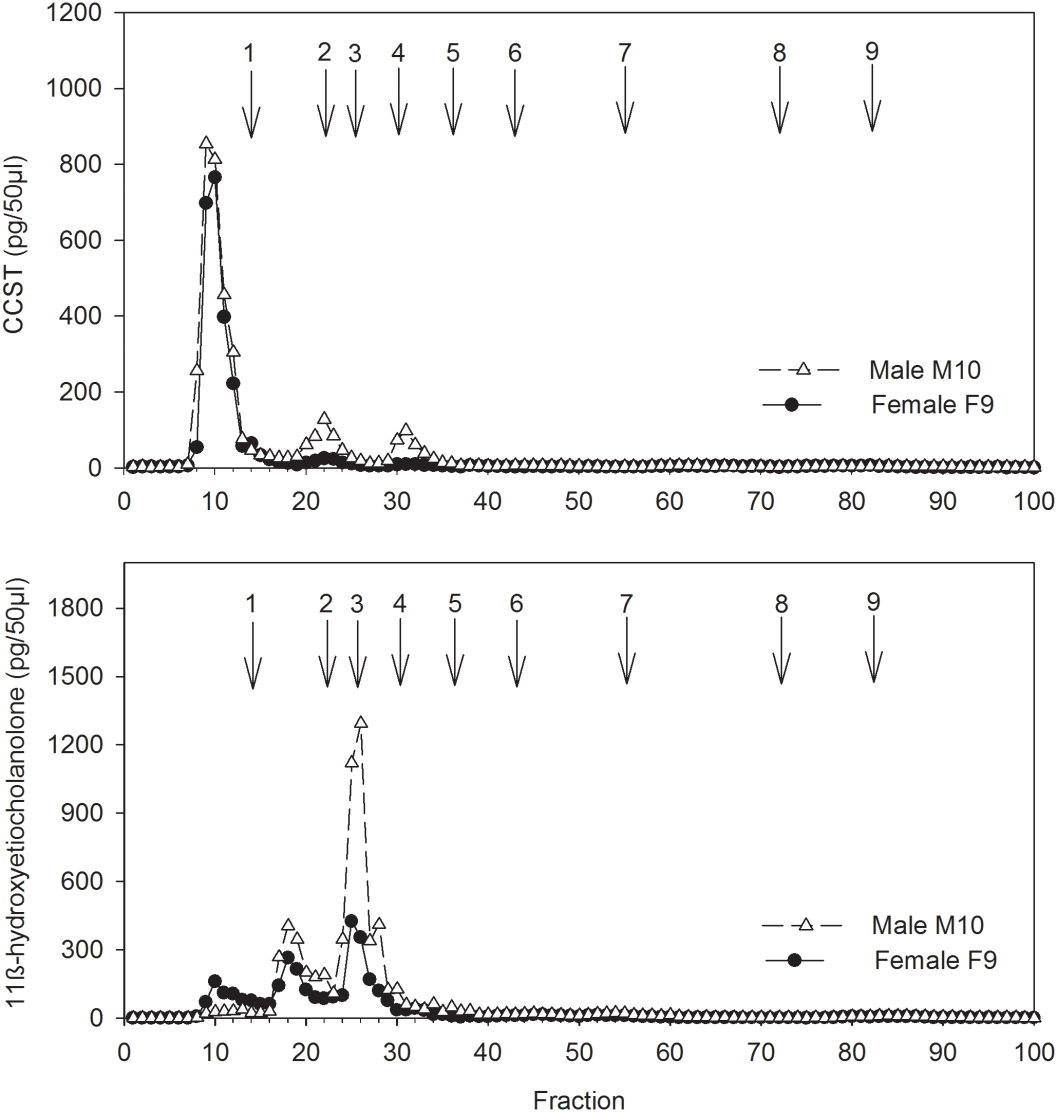
HPLC profiles of immunoreactivity detected with the CCST (top) and 11β-hydroxyetiocholanolone EIA (bottom) for a male and a female meerkat. Samples tested were those that showed peak GC metabolite concentrations after the ACTH challenge. Arrows and numbers show the elution positions of associate reference standards: 1) cortisol (fractions 14–15), 2) corticosterone (22), 3) 11β-hydroxyetiocholanolone (25), 4) 11-oxoetiocholanolone (30), 5) 5β-androstane-3,11,17-trione (36), 6) testosterone (43), 7) androstendione, dehydroepiandrosterone (55–56), 8) epiandrosterone, 5β-DHT, 5b-androstane-3β-ol-17-one (72), 9) 5β-androstane- 3α-ol-17-one (82).

HPLC analysis indicated that in both GC assays the vast majority of immunoreactivity (>90%) was detected as three distinct peaks between fractions 9 and 32 (Fig 3)–positions where cortisol metabolites in our HPLC system elute [68]. The positions of the three peaks, however, differed between the two assays. While the major peak of immunoreactivity in the CCST assay was found at fraction 10, representing an unknown metabolite of high polarity, the major peak measured by the 11β-hydroxyetiocholanolone EIA was found at fraction 25, the elution position of authentic 11β-hydroxyetiocholanolone, indicating a high abundance of this metabolite of cortisol in the feces of meerkats. The presence of negligible amounts of immunoreactivity measured after fraction 40 (positions where certain potentially cross-reacting gonadal and adrenal androgen metabolites elute [68, 69], suggests a low degree of co-measurement of these androgens in our assay (Fig 3). For both assays, HPLC profiles were similar between males and females in terms of both number and elution position (i.e. characteristics) of metabolites measured.

### Effect of sample soil contamination on FGCM values and ACTH response characteristics

**Fig 4:**
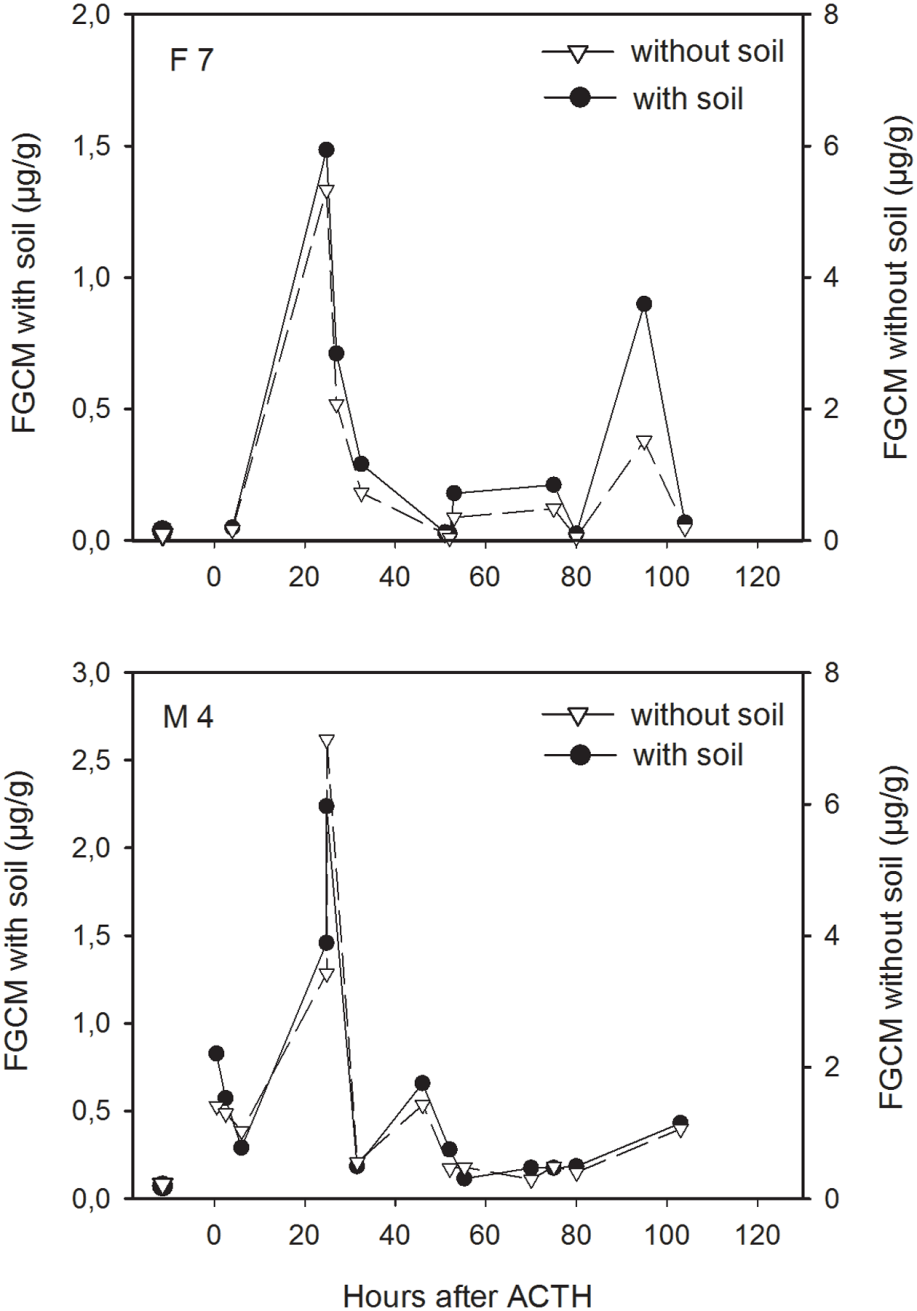
Representative FGCM (11β-hydroxyetiocholanolone) profiles of a female (top) and male (bottom) meerkat after administration of ACTH in samples uncorrected for soil contamination (with soil) and those corrected for soil content (without soil). Data points before time 0 indicate median ± SE concentrations of pre-treatment samples collected 5–8 days before ACTH injection. Profiles for CCST showed a similar degree of correspondence between the two conditions (see text).

**Fig 5:**
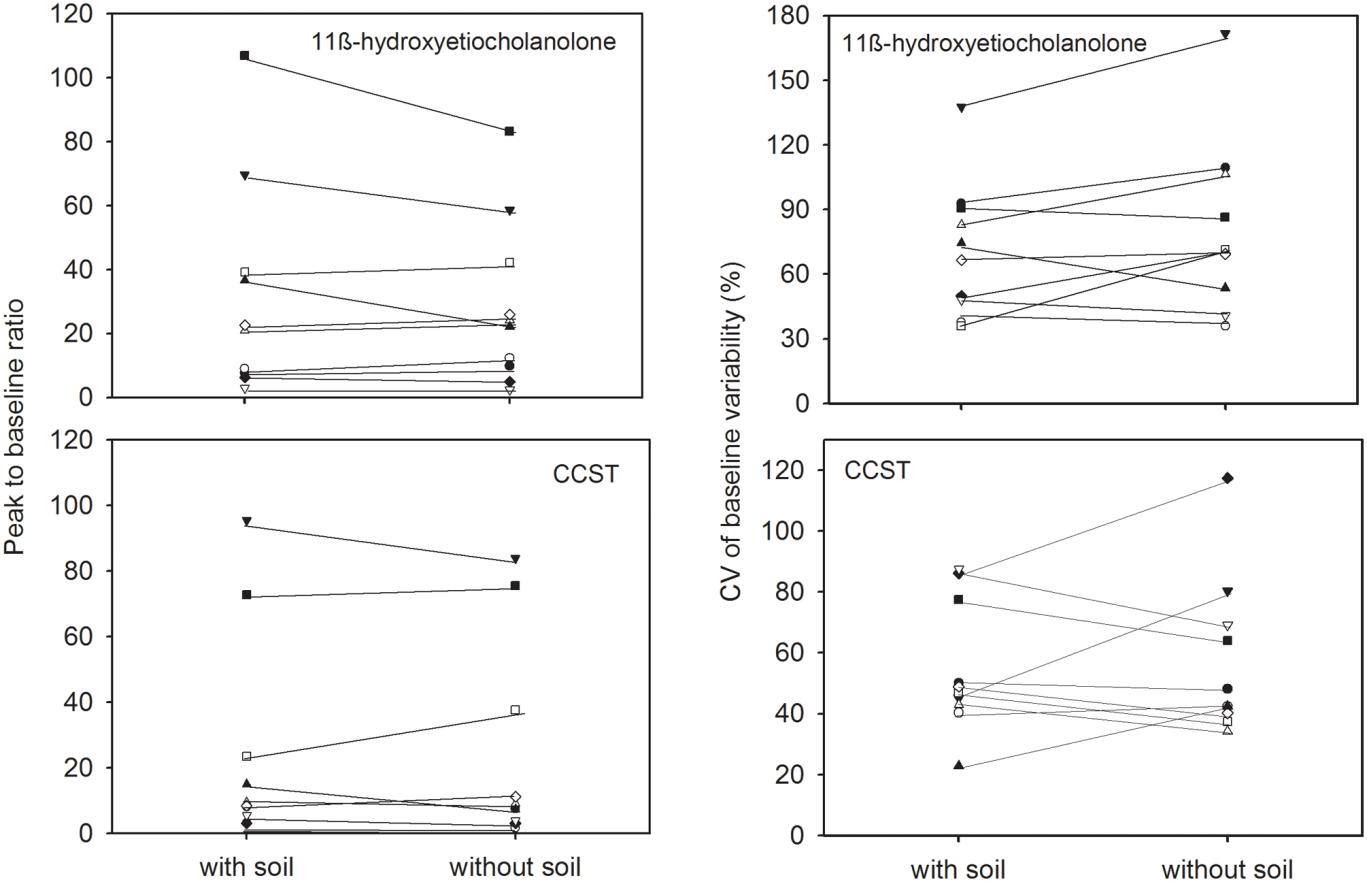
Magnitude of 11β-hydroxyetiocholanolone and CCST elevation to ACTH (peak to baseline ratio; graphs on the left) and variability in 11β-hydroxyetiocholanolone and CCST (pre-treatment) baseline values (coefficients of variation (CV) of baseline variability; graphs on the right) in samples uncorrected for soil contamination (with soil) and those corrected for soil content (without soil) in 10 meerkats.

Across all fecal samples tested (n = 182), soil contamination ranged from as low as 5.4% to as high as 81.2% of each sample’s total mass. Across animals, on average, 59.6% (range 55.1–65.2%) of the total fecal sample mass extracted was composed of soil. Removing the soil from the samples and expressing FGCM concentrations per soil-free fecal mass rather than per total mass extracted fecal material generally did not change response patterns to ACTH (Fig 4) nor did it affect the variability in pre-treatment basal levels and magnitude of FGCM elevation to ACTH significantly (Fig 5). Specifically, FGCM profiles uncorrected and corrected for soil contamination correlated strongly in each of the 10 animals tested, with mean r- values of 0.90 (range: 0.69 to 1.0) for the CCST and 0.95 (range: 0.88 to 1.0) for the 11β-hydroxyetiocholanolone measure. The correlations were significantly higher for the 11β-hydroxyetiocholanolone compared to the CCST measure (W = 1, p = 0.021). Correction for soil contamination did not significantly affect variability (expressed as CV-value) of basal FGCM levels for neither 11β-hydroxyetiocholanolone (median CV ± SE with soil: 70.7 ± 9.9%; without soil: 70.4 ± 12.7%; W = 15, p = 0.232) nor CSST (with soil: 48.0 ± 6.8%; without soil: 45.3 ± 8.2%; W = 26.5, p = 0.959). Furthermore, the magnitude of peak FGCM elevation to ACTH did not differ significantly using FGCM values corrected for soil contamination compared to those uncorrected for soil contamination for either FGCM measure (11β-hydroxyetiocholanlone: median ± SE with soil: 21.8 ± 10.5; without soil: 23.1 ± 8.3, W = 34, p = 0.557; CCST: 8.9 ± 10.3; without soil: 8.3 ± 9.8, W = 31, p = 0.770).

### FGCM response to subordinate male eviction

FGCM levels of individuals attacking a single male differed significantly between prior, during, and post the event (Friedman: *X*^2^ = 7.6, exact p = 0.024, n = 5; Fig A in S1 File), with median FGCM concentrations being almost 6-fold higher during the attack (median ± SE: 1.102 ± 0.175 μg/g) compared to pre-attack levels (median ± SE: 0.141 ± 0.065 μg/g), and 4-fold higher compared to post-attack levels (median ± SE: 0.185 ± 0.044 μg/g). Pre- and post-attack FGCM levels did not differ significantly (Wilcoxon rank-test: Z = 0.135, exact p = 1.0, n = 5). Using the full data set (n = 10) to compare during- and post- attack levels confirmed that FGCM levels within 48 hours of the attack were significantly higher than levels in samples collected about 1 month after the attack (median ± SE; within 48 hours: 0.810 ± 0.145 μg/g; 1 month after attack: 0.202 ± 0.028 μg/g; Wilcoxon rank-test: Z = 2.599, exact p = 0.006, n = 10). Averaged FGCM levels of the evicted male (n = 2 samples; median ± SE: 1.737 ± 0.099 μg/g) were the highest within 48 hours of the attack (Fig A in S1 File).

### Effect of sex, age and time of day on FGCM values

Overall, males and females did not differ significantly in their basal FGCM levels (median ± SE; males: 0.17 ± 0.05 μg/g, females: 0.13 ± 0.09 μg/g; t = 1.04, p = 0.316; Table 2). Age (in months) was not significantly associated with basal FGCM values although there was a tendency for FGCM’s to increase with age (LMM: t_13.65,123_ = 1.78, p = 0.09). We found a significant effect of time of day on basal FGCM, with levels being significantly higher in the morning (8-11AM) period compared to midday (11:30-2PM) (LS Means: AM-MD: t = 2.60, p = 0.02) and showed a tendency to be higher than in the evening (3-6PM) period (LS Means: AM-PM: t = 1.01, p = 0.07). Midday and evening FGCM levels did not differ (LS Means: MD-PM: t = 0.224, p = 0.32, Fig B in S1 File).

## Discussion

Monitoring of glucocorticoid excretion patterns of animals via fecal analysis has become an established method in the study of stress physiology of wild populations due to its non-invasive nature (e.g. [15]). In the current study, we assessed the suitability of two group-specific cortisol metabolite assays in comparison to a formerly used corticosterone (CCST) assay for tracking adrenocortical activity from fecal samples of meerkats, and examined the potential influence of intrinsic (age, sex) and extrinsic (time of day, contamination of feces by soil) factors on fecal glucocorticoid measurements. We show that all three assays tested were able to detect the glucocorticoid response to pharmacological stimulation of the adrenal gland by ACTH. We demonstrate, however, that the group-specific measurement of metabolites of cortisol using an 11β-hydroxyetiocholanolone assay produced more consistent results and presents greater biological sensitivity than the CCST measurement previously used to study meerkat stress physiology [52]. We also show that time of day but not age or sex influence FGCM excretion and that soil contamination of fecal samples does not affect FGCM patterns to a significant extent. Our study therefore provides important new information for researchers interested in using FGCM analysis to monitor the stress physiology in captive and wild meerkats, and highlights the importance of conducting extensive assay validations to gain a more comprehensive understanding of any species’ adrenocortical function.

### ACTH challenge and validation of FGCM measurements

All three EIAs used detected the predicted FGCM response to the ACTH challenge reliably in both male and female meerkats. Regarding the CCST measurement, our results confirm and extend previous findings from a study of Young et al. [52] conducted on a limited number of wild-living animals (n = 4) using relatively infrequent samples. Our study thus provides confirmation for the validity of the fecal CCST measurement as an indicator of adrenocortical function applied in previous studies on stress physiology in meerkats [47, 52, 73]. Compared to the CCST measurement, however, the measurement of immunoreactive 11β-hydroxyetiocholanolone, a major metabolite of cortisol in mammals (e.g. [68, 69]), presented overall a much stronger response to the ACTH challenge (as well as to the control water injections), indicating an enhanced biological sensitivity of this assay for detecting changes in HPA axis activity [21, 74–76] in meerkats. A more sensitive assay enables smaller-scale differences in glucocorticoid production to be detected [74, 76], making the 11β-hydroxyetiocholanolone assay potentially superior over the CCST assay, particularly when stress-related changes in FGCM levels are of lower magnitude (c.f. [76]). Our findings that peak responses obtained with the 11β-hydroxyetiocholanolone assay were more consistent between individuals and had a much less variable time lag compared to the CCST assay are in support of our conclusion that the 11β-hydroxyetiocholanolone assay may track adrenocortical activity in meerkats more accurately than the CCST EIA, a finding also reported in several other studies [77] (but see [21, 65, 68, 72]).

Moreover, HPLC indicated 11β-hydroxyetiocholanolone to be abundant in the feces of meerkats, as reported for other species (e.g.[62, 68, 69, 78]). By contrast, only a small portion of the immunoreactivity measured by the CCST assay could be ascribed to native CCST with the assay mainly reacting with an unknown compound of high polarity. Whether this polar substance is a metabolite of cortisol as is 11β-hydroxyetiocholanolone or whether its excretion is just correlated with GC production is unclear. Solving this question would require more detailed studies on cortisol metabolism in meerkats using a radioinfusion study in combination with HPLC (e.g. [79]). Importantly, we did not detect any immunoreactivity after fraction 40 with either assay, suggesting that there was no co-measurement of particular androgens potentially cross-reacting in the FGCM assays as a result of structural similarities between fecal metabolites of cortisol and testosterone [31, 68, 69, 77].

While it is valuable to show that a selected FGCM measure is able to pick up a pharmacologically induced increase in cortisol, it is similarly important that the measure is sensitive enough to detect the physiological stress response to more natural biological stressors encountered in the normal life of an animal [80]. We could demonstrate this for our 11β-hydroxyetiocholanolone assay by showing a marked elevation in FGCM levels in response to agonistic interactions among group members leading to the eviction of a subordinate male (see also [52]). Moreover, the very short-term stressor of applying pure water injections also triggered significant elevations in 11β-hydroxyetiocholanolone (and CCST) excretion. This finding demonstrates that the injection procedure itself induced a stress response (see [81] for mice), even without having restrained the individuals and albeit them being well habituated to human contact and handling. Researchers should consider this when studying the stress physiology of wild meerkats where handling animals for various reasons is not uncommon (e.g. [26, 82, 83]).

### Time course of FGCM excretion and effect of time of day, age and sex on FGCM levels

The use of captive animals and collection of multiple daily samples per individual allowed us to assess the time course in FGCM excretion, examine potential age effects and sex differences as well as investigate circadian excretion patterns of FGCM, contributing important knowledge to our understanding of patterns of glucocorticoid excretion in meerkats. The average (i.e. median) time lag between stressor (ACTH challenge, water injections) and detection of increased 11β-hydroxyetiocholanolone levels in feces was 24–25 hours which was at the lower end of the 24–48 hour time lag reported for the FGCM measure by Young and colleagues [52]. A time lag of about one day as found here is also in line with FGCM lag times reported for many other species [84]. Generally, delay times between circulating GCs and their metabolites in feces vary from as short as 2–3 hours (Tufted capuchin monkeys, *Cebus apella*: [65]) to as long as 48 hours (pigs, *Sus domesticus*: [85]), mainly as a result of species-specific gut passage times [32].

We found a marked effect of time of day in FGCM excretion with significantly higher basal FGCM levels in morning samples compared to samples collected later during the day. Diurnal variation in FGCM has been reported for other species, in particular in those that defecate at relatively high rates (i.e. providing a higher temporal resolution in the feces; e.g. [72, 80, 81]) and as such our data would support these findings as meerkats also defecate relatively frequently over the day. Nevertheless, our result contrasts, with a report from field studies on meerkats (e.g. [22]) where no diurnal variation in FGCM was detected. This difference likely results from two factors. First, differences in eating patterns between captive and wild meerkats, as wild individuals forage continuously on small prey items throughout the day while our captive meerkats are fed three times daily, probably result in different defecation rates. Second, the higher biological sensitivity and time-frame consistency of the 11β-hydroxyetiocholanolone assay (used here) compared to the less sensitive CCST assay used by Voellmy and colleagues [22] likely facilitated the identification of this diurnal effect. For these reasons, we recommend that in future studies time of day be considered as a potential factor influencing FGCM levels in meerkats. We did not find differences in basal FGCM levels or in response patterns to the ACTH challenge between males and females. We are confident that this is a valid result given that our HPLC data indicated that the immunoreactive compounds measured in our assays were similar between the sexes. Thus, FGCM levels reported here seem to not be biased by cross-reactivity of the antibody with particular sex-specific hormone metabolites or by sex-specific differences in cortisol metabolism (see [34] for mice), both of which representing potential major concerns with respect to data interpretation [39, 86]. Absence of a sex-specific effect on FGCM levels as found here for meerkats has also been reported for other mammals such as California mice, *Peromyscus californicus* [87], howler monkeys, *Alouatta pigra* [72, 88] and spider monkeys, *Ateles hybridus* [72]. As with sex, age appeared to have no significant effect on FGCM excretion in meerkats. However, this finding needs to be treated with caution since our study was restricted to adult individuals with a relatively limited age range. Moreover, our results for an age effect are potentially confounded by a possible rank effect on FGCM levels as in meerkats dominant individuals are usually also the eldest in each sex [83, 89].

### Effect of soil contamination on FGCM response characteristics

As meerkats naturally inhabit arid, sandy regions of southern Africa and, when in captivity, are commonly kept in semi-natural enclosures with sandy substrates, we assessed the potential effects of sample contamination by sand on the pattern of FGCM levels. Although we grossly removed the sand from the outer layer of the feces, samples were still composed of 50–60% sand, which can potentially influence the non-biological variation in the FGCM measure [67]. However, correcting FGCM concentrations for sand content did not significantly change FGCM pattern across samples. Specifically, FGCM levels in the samples before and after sand content “correction” correlated strongly and sand-corrected samples did not show higher magnitudes of peak response to ACTH or lower variability of basal levels. Our simple approach to rubbing off the sand from the sample prior to pulverization and extraction thus appeared to have been effective enough to reduce potential FGCM variation due to sand contamination [67] to an extent that did not impact the results a great deal. Thus, our results show that future studies investigating FGCM excretion in meerkats do not need to apply extensive methods (such as fecal combustion) to remove the soil from samples prior to hormone analyses [67], simplifying procedures and lessening time and cost constraints.

Apart from fecal contamination, other environmental factors have been shown to affect both FGCM excretion and recovery in mammals. For example, seasonal changes in rainfall affect FGCM excretion in a number of species (baboons, *Papio cynocephalus*: [90] white-tailed deer, *Odocoileus virginianus*: [91]) as do photoperiod [42] and temperature [92]. Moreover, FGCM recovery can be affected if fecal samples are not collected immediately after deposition [93]. To what extent these factors influence excretion patterns and concentrations of FGCM in meerkats is unknown and thus remains to be examined.

In conclusion, the work presented here provides an extensive and robust methodological validation of the assessment of adrenocortical activity in meerkats via analysis of FGCM, extending previous work [52] substantially. The methods and findings described here will facilitate the study of links between glucocorticoid production, welfare, social behavior, dispersal decisions and communication in an already well-understood animal system, in the wild and in captivity. As meerkats are one of the best studied species in the study of the evolution of mammalian societies and cooperative breeding, such knowledge may also provide important insights into the mediation of social behavior. Furthermore, the results presented here may potentially be extrapolated to other social mammals and cooperative breeding species that, for a variety of reasons, may be more difficult to study, especially in their natural habitat. Last but not least, we hope our work will also contribute to accurate monitoring and improvement of meerkat welfare in zoos and other captive colonies.

## Supporting Information

S1 File Group FGCM response (median ± SE, μg/g) to a natural attack on a group member (ZIM005) that resulted in its permanent eviction. The victim showed the greatest FGCM response to the event. Group FGCM levels returned to baseline levels after the male was removed from the colony. “Within 48 h” represent FGCM levels measured within 2 days after the attack on M5 took place. ** = p < 0.01 (Fig A). Average (median ± SE) baseline FGCM levels (μg/g) in fecal samples deposited in the morning (AM), at midday (MD) and late afternoon (PM), as measured with the 11β-hydroxyetiocholanolone assay. N = 128 fecal samples from 13 individuals. * = p < 0.05 (Fig B).
(DOCX)
