# Supplementary material for: Validation of a Fecal Glucocorticoid Assay to Assess Adrenocortical Activity in Meerkats Using Physiological and Biological Stimuli
Source: PLoS One. 2016 Apr 14;11(4):e0153161. doi: 10.1371/journal.pone.0153161 (PMC4831846; doi:10.1371/journal.pone.0153161)
Supplement: S1 File — The victim showed the greatest FGCM response to the event. Group FGCM levels returned to baseline levels after the male was removed from the colony. “Within 48 h” represent FGCM levels measured within 2 days after the attack on M5 took place. ** = p < 0.01 (Fig A). Average (median ± SE) baseline FGCM levels (μg/g) in fecal samples deposited in the morning (AM), at midday (MD) and late afternoon (PM), as measured with the 11β-hydroxyetiocholanolone assay. N = 128 fecal samples from 13 individuals. * = p < 0.05 (Fig B). (DOCX) [file pone.0153161.s001.docx]

**Supporting Information:**


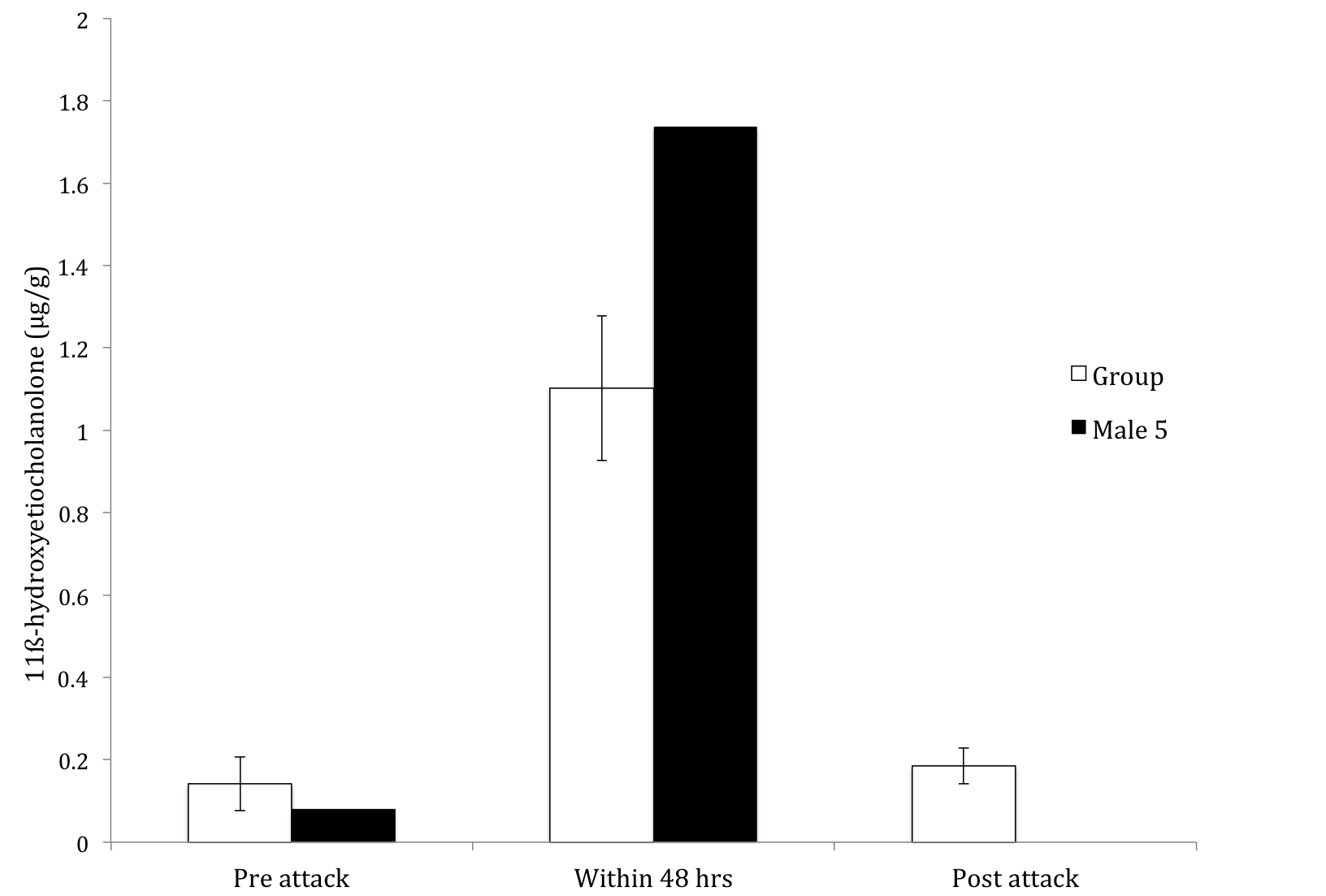


**

**Figure A**: Group FGCM response (median ± SE, µg/g) to a natural attack on a group member (M5) that resulted in its permanent eviction. The victim showed the greatest FGCM response to the event. Group FGCM levels returned to baseline levels after the male was removed from the colony. “Within 48 h” represent FGCM levels measured within 2 days after the attack on M5 took place. ** = p < 0.01.

**Figure B:** Average (median ± SE) baseline FGCM levels (µg/g) in fecal samples deposited in the morning (AM), at midday (MD) and late afternoon (PM), as measured with the 11ß-hydroxyetiocholanolone assay. N = 128 fecal samples from 13 individuals. * = p < 0.05.
